# NAPOK: Nationales Register zur Analyse (non-)invasiver Therapieverfahren des postpunktionellen Kopfschmerzes – Protokollpublikation

**DOI:** 10.1007/s00101-025-01512-x

**Published:** 2025-02-11

**Authors:** Philipp Helmer, Peter Kranke, Jan Niklas Thon, Antonia Helf, Patrick Meybohm, Markus A. Weigand, Benedikt H. Siegler, NAPOK Steering Committee, Karin Becke-Jakob, Susanne Greve, Philipp Helmer, Peter Kranke, Markus A. Weigand, Benedikt H. Siegler, Jan Wallenborn, Hinnerk Wulf, Wolfgang Zink

**Affiliations:** 1 Klinik und Poliklinik für Anästhesiologie, Intensivmedizin, Notfallmedizin und Schmerztherapie, Universitätsklinikum Würzburg, Oberdürrbacher Straße 6, 97080 Würzburg, Deutschland; 2 Medizinische Fakultät Heidelberg, Klinik für Anästhesiologie, Universität Heidelberg, Im Neuenheimer Feld 420, 69120 Heidelberg, Deutschland

## Hintergrund & Hypothesen

Der Postpunktionskopfschmerz („postdural puncture headache“, PDPH) kann als Komplikation sowohl infolge einer gezielten als auch einer ungewollten Punktion der harten Hirnhaut (Dura mater) auftreten. Gemäß der internationalen Kopfschmerzklassifikation wird diese sekundäre Zephalgie durch einen erniedrigten Liquordruck ausgelöst [1]. Dieser kann nach einer Vielzahl medizinischer Maßnahmen, beispielsweise als Komplikation einer rückenmarknahen Regionalanästhesie, nach Anlage eines Spinalkatheters, nach diagnostischen oder therapeutischen Lumbalpunktionen oder infolge der Eröffnung der Dura mater im Rahmen neurochirurgischer Interventionen auftreten.

Je nach Intervention variieren die berichteten PDPH-Inzidenzen zwischen 0,3 und 1,7 % nach Spinalanästhesie, 6 % nach Lumbalpunktion bis hin zu > 80 % nach ungewollter Duraverletzung während der Anlage eines Periduralkatheters [2–5]. Per definitionem treten PDPH-assoziierte Symptome innerhalb von 5 Tagen nach Durapunktion auf [1]. Ein früher Symptombeginn (48–72 h nach Punktion) ist häufig, allerdings sind auch Fälle mit einer deutlichen Latenz beschrieben [6–8]. Der meist als dumpf-pochend beschriebene Schmerz ist häufig frontal, temporoparietal oder okzipital lokalisiert, kann in Nacken, Schultern oder Rücken ausstrahlen und nimmt charakteristischerweise in aufrechter Körperposition zu, wobei diese Lageabhängigkeit auch fehlen kann und umgekehrt das Vorliegen eines PDPH nicht beweist [9]. Der Kopfschmerz kann isoliert oder mit Begleitsymptomen (u. a. Doppelbildersehen, Tinnitus, Fotophobie, Schwindel, Nausea und Dysakusis) auftreten [10]. Über das Akutereignis hinaus kann ein PDPH mit erheblichen physischen und psychischen (Langzeit‑)Komplikationen einhergehen.

Die Behandlungsmöglichkeiten reichen von konservativen Maßnahmen über medikamentöse Therapien bis hin zu invasiven Verfahren wie dem epiduralen Blutpatch (EBP) [11], welcher nach wie vor den therapeutischen Goldstandard bei Versagen medikamentös-konservativer Maßnahmen darstellt [12]. Eine Besserung kann bei 50–80 % der mittels EBP behandelten Patient:innen erzielt werden [13]. Gleichwohl ist die Applikation eines EBP auch mit z. T. schwerwiegenden Komplikationen assoziiert. Zu diesen gehören Infektionen, Blutungskomplikationen oder neurologische Auffälligkeiten (u. a. aseptische Meningitis, Arachnoiditis) [9, 14, 15]. Zudem ist eine erneute Duraverletzung bei EBP-Anlage möglich.

Vor diesem Hintergrund und der nicht selten ablehnenden Haltung der Patient:innen gegenüber einer erneuten Punktion sind alternative Therapieoptionen von zunehmendem Interesse. Weniger invasive Verfahren wie die therapeutische Anwendung von Lokalanästhetika bieten potenzielle Vorteile, sind jedoch bislang nur unzureichend untersucht [11]. Insbesondere die aus der selektiven Betrachtung von Subkollektiven resultierenden geringen Fallzahlen tragen dazu bei, dass ein umfassender Vergleich aller eingesetzten Therapieoptionen des Postpunktionskopfschmerzes noch aussteht. Ziel des hier beschriebenen Projekts ist daher der Aufbau eines Registers zur Erfassung und Analyse sämtlicher Verfahren zur Behandlung des PDPH. Auf Basis der gesammelten Daten ist perspektivisch neben der Beantwortung epidemiologischer Fragestellungen u. a. auch die Untersuchung und Evaluation der zur PDPH-Therapie eingesetzten Verfahren in Bezug auf Wirksamkeit sowie Komplikationen geplant.

## Details der Studie

NAPOK ist eine prospektive, multizentrische, nichtinterventionelle Registerstudie. Es werden Daten von Patienten mit PDPH aus Krankenhäusern in ganz Deutschland unabhängig von deren Versorgungsstufe erfasst. Das Register sammelt strukturierte Daten zu konservativen, medikamentösen, invasiven sowie experimentellen Behandlungsverfahren, einschließlich deren Erfolgsraten und möglicher behandlungsassoziierter Komplikationen.

Um eine möglichst umfassende Studienpopulation abzudecken, sind als Einschlusskriterien lediglich ein Alter ≥ 18 Jahre, das Vorliegen eines PDPH (unabhängig von der Ursache), Einwilligungsfähigkeit sowie die Erteilung einer schriftlichen Einwilligungserklärung definiert. Die Teilnahme ist freiwillig; ein Widerruf der Zustimmung ist jederzeit möglich.

Die im Register erfassten Daten und Parameter werden in Tab. 1 zusammengefasst. Zur Erfassung des mittel- und langfristigen Behandlungsergebnisses erfolgen fakultativ nach 3 und 18 Monaten telefonische Follow-up-Befragungen. Nach Pseudonymisierung im jeweiligen Zentrum erfolgt die Datenübermittlung mithilfe elektronischer Erfassungsbogen (Research Electronic Data Capture, REDCap®; REDCap platform and consortium, Vanderbilt University, Nashville, USA).

**Tab. 1:** Zusammenfassung erhobener Parameter

Kategorie	Erfasste Parameter (Auszug)
Personenbezogene Daten (Verbleib im jeweiligen Zentrum)	Vorname, Nachname, Geburtsdatum
Zentrums-/abteilungsbezogene Daten (Erhebung geplant)	Versorgungsstufe, sofern zutreffend jährliche Geburtenzahl, Zahl und Art von durchgeführten Regionalanästhesien, Spinalkatheteranlagen und/oder Lumbalpunktionen pro Jahr
Allgemeine demografische Daten	Alter, Größe, Gewicht
Daten zur stationären Behandlung	Dauer der stationären Behandlung, ggf. Dauer des Aufenthalts auf der Intensivstation, Notwendigkeit einer erneuten PDPH-bedingten stationären Aufnahme oder ambulanten Behandlung
Schwangerschaftsassoziierte Daten (sofern zutreffend)	Gravidität/Parität, Schwangerschaftswoche zum Zeitpunkt der Entbindung, Geburtsmodus (Spontanpartus, Sectio caesarea), Vorliegen einer Mehrlingsschwangerschaft, geburtshilfliche Komplikationen
Daten zu Regionalanästhesie, Spinalkatheteranlage oder Lumbalpunktion	Art der Punktion (SPA, PDA, Spinalkatheter, Lumbalpunktion) sowie technik- und anwenderbezogene Details (u. a. Größe der verwendeten Nadel, Punktionshöhe, Anzahl der Punktionsversuche, Ausbildungsstand der/des Punktierenden, Komplikationen während der Anlage)
Daten zur Diagnostik des PDPH	Zeitpunkt der PDPH-Diagnose (Zeit zwischen Regionalanästhesie und Beginn der Symptomatik sowie der PDPH-Diagnose), Schmerzqualität und -intensität sowie Art und Dauer weiterer PDPH-Symptome, Daten zur PDPH-induzierten Einschränkung der Belastbarkeit und Lebensqualität, differenzialdiagnostisches Vorgehen, Bildgebung
Daten zur Therapie des PDPH	Art und Dauer der durchgeführten Therapie (konservativ-medikamentös, therapeutische Lokalanästhesie, Anlage eines EBP, weitere neuartige Ansätze etc.) sowie Daten zu deren Wirksamkeit, Nachsorge und potenziellen Komplikationen
Daten zu Komorbiditäten	Vorhandensein von Risikofaktoren für die Entwicklung eines PDPH, sonstige Komorbiditäten
Daten zu Komplikationen	Auftreten von Infektionen, neurologischen oder kardiopulmonalen Komplikationen oder anderen unerwünschten Ereignissen im Zusammenhang mit der Therapie

*EBP* epiduraler Blutpatch; *PDA* Periduralanästhesie; *PDPH* Postpunktionskopfschmerz; *SPA* Spinalanästhesie

Das NAPOK-Register ermöglicht über die Erfassung sämtlicher PDPH-Therapieformen u. a. erstmals umfassende Vergleiche hinsichtlich der Wirksamkeit unterschiedlicher Maßnahmen. Als Besonderheit bietet es die Möglichkeit, auch neue/experimentelle Therapieansätze strukturiert zu erfassen und damit vergleichbar zu machen. Neben der Evaluation der Effektivität therapeutischer Maßnahmen ermöglicht NAPOK zudem, seltene Ereignisse zu ermitteln, die i. R. kleinerer Studien ggf. nicht erfasst werden (z. B. behandlungsassoziierte Komplikationen). Zukünftige Forschungsprojekte, welche auf diese Datengrundlage zurückgreifen, besitzen daher das Potenzial, das klinische Vorgehen bei Auftreten dieser interdisziplinär relevanten Komplikation weiter zu optimieren.

## Mitwirkung

Wenn Sie rückenmarknahe Anästhesieverfahren in Ihrer Klinik durchführen, unabhängig von Schwerpunkt oder Versorgungsstufe, laden wir Sie herzlich ein, Teil unseres Registers zu werden. Der zeitliche Aufwand pro Patienteneinschluss beläuft sich inklusive Aufklärungsgespräch, Dateneingabe und telefonischer Follow-up-Befragung nach ersten Erfahrungen auf ca. 45–60 min. Wenn wir Ihr Interesse geweckt haben, wenden Sie sich bitte an den Studienleiter Priv.-Doz. Dr. med. Benedikt H. Siegler, Universität Heidelberg.

## Ethik

Das Register folgt den Prinzipien der Deklaration von Helsinki in ihrer aktuellen Fassung. Zustimmende Bewertungen nach berufsrechtlicher Beratung durch die Ethikkommission Heidelberg (Erstvotum AZ: S‑732/2022) sowie die zuständigen Stellen der bislang beteiligten Zentren liegen vor. Voraussetzung für die zukünftige Auswertung oder Nutzung der im Register gesammelten Daten ist eine auf das entsprechende Vorhaben bezogene Beratung durch die jeweils zuständige Ethikkommission.

## Meilensteine

Das positive Erstvotum der federführenden Ethikkommission in Heidelberg erfolgte im November 2022. Nach Implementierung der notwendigen Infrastruktur startete die Patientenrekrutierung im Dezember 2023. Bei einer geplanten Mindestlaufzeit von 5 Jahren erfolgt nun die Akquise weiterer Zentren, um ein nationales Register zu etablieren. Aktuell (Stand Februar 2025) befinden sich 4 Universitätskliniken in der aktiven Rekrutierungsphase; weitere 22 interessierte Zentren aus insgesamt 10 Bundesländern befinden sich in der Vorbereitungsphase. Im Rahmen von regelmäßigen Zwischenauswertungen sollen die Datenqualität überprüft und erste Ergebnisse zu Effektivität und Komplikationsrate der verschiedenen Therapieansätze ausgewertet werden.

## Studiengruppe/Expertise

### Studienleiter.

Priv.-Doz. Dr. med. Benedikt H. Siegler
